# Scientific Hysteria

**Published:** 1879-07

**Authors:** 


### ARTICLE III.

### *Scientific Hysteria.*

No stronger illustration of the power and worth of truth is seen than in the constant simulation of the truth. The highest achievement of man is to so imitate the real as to delude the observer, and defy, at least for a time, detection. The desire for prominence, the passion for conspicuousness, the anxiety to be before the public and to gain reputation for scientific attainments, leads not a few into invention in the place of fact, and develops a cheap notoriety in the place of real reputation, and occasionally brings on a diseased condition which may not inappropriately be denominated "Scientific Hysteria." And a more inveterate, troublesome and altogether unmanageable disease comes not within the range of knowledge of man; incurable and desperately chronic. It is said that hysteria simulates any and all diseases known to the medical profession, and is so clever in imitation as to entirely delude the novice; and in scientific hysteria, nothing in the way of science is developed but is made the occasion by the victim of this malady of a new and alarming fit. Is a new idea advanced, the diseased man has a newer one still, far eclipsing the discoverer; is an advance made, the hysteric is more advanced ; is investigation attempted, the sufferer steps forth with "Behold my work in that department." Redundant in words, he covers up the ideas he may have with such an effervescing froth of language as to perplex those who listen to him, and puzzle the hearer or reader as to whether the subject matter was rightly announced. Valuble and loud, he bears down opposition, and replies to criticism with a torrent of gush, until submission is yielded as the only way of escape from his deluge of words.

True science is quiet, calm, unassuming, without pretence or bluster, and her advocates and apostles can calmly afford to await, if need be, the verdict of ages. But the hysterical scientist in his wild raving, is furious with all who agree not with him instantly. "Verily this is the truth, and you who do not instantly accept it are blind, foolish, and wicked." "I have discovered, and all that has gone before which contradicts or disagrees with my discovery is not simply a mistake, but falsity itself, and its advocates asses." If he is mildly asked for the evidences of his discoveries, he raves, storms, calls on supernatural help, and drowns the questioner with a deluge of words, complex sentences and high-sounding technecalities which exhaust the speaker and quiet the interlocutor.

Those who have not this disease should be truly grateful. Doubtless it is connate, impossible of cure. But whether acquired or inherited the subject of it is truly a source of terror to his friends as much, if not more than to his foes. Science is great, the truth itself is science, and her apostles are and should be entitled to not only respect but reverence; but for your hysterical scientist, with his ravings and stormings and declarations of discovery, deliver us. Doubtless nothing can be done with such cases, they cannot be locked up nor even locked out; they are ever on the floor, ever before the public, but if this may be classed among the list of "preventable diseases," if there is chance of avoiding it, let us who see it in others, study to avoid it ourselves as a plague.

